# RNA-Binding Protein HuR Regulates Rac1 Nucleocytoplasmic Shuttling Through Nucleophosmin in the Intestinal Epithelium

**DOI:** 10.1016/j.jcmgh.2019.06.002

**Published:** 2019-06-10

**Authors:** Lan Liu, Lan Xiao, Hee K. Chung, Min S. Kwon, Xiao-Xue Li, Na Wu, Jaladanki N. Rao, Jian-Ying Wang

**Affiliations:** 1 Cell Biology Group, Department of Surgery, University of Maryland School of Medicine, Baltimore, Maryland; 2 Research Service, Baltimore Veterans Affairs Medical Center, Baltimore, Maryland; 3 Department of Pathology, University of Maryland School of Medicine, Baltimore, Maryland

**Keywords:** RNA-Binding Proteins, GTP-Binding Proteins, IECs, Nucleocytoplasmic Shuttling, Conditional Deletion, AdHuR, recombinant adenoviral plasmids containing human HuR complementary DNA, ATF, activating transcription factor, Cdc42, Cell division control protein 42, cDNA, complementary DNA, Chk2, Checkpoint kinase 2, COS7, Fibroblast like cell line COS7, C-siRNA, control small interfering RNA, GTP, guanosine triphosphate, HeLa, Human cervical cancer cell line, IEC, intestinal epithelial cell, IE-HuR^-/-^, intestinal epithelial tissue-specific HuR knockout, lncRNA, long noncoding RNA, mRNA, messenger RNA, NPM, nucleophosmin, RBP, RNA-binding protein, siHuR, small interfering RNA targeting HuR, siNPM, small interfering NPM, 3’-UTR, 3’-untranslated region

## Abstract

**Background & Aims:**

The mammalian intestinal epithelium is a rapidly self-renewing tissue in the body, and its homeostasis is tightly regulated via well-controlled mechanisms. The RNA-binding protein HuR is essential for maintaining gut epithelial integrity, and targeted deletion of HuR in intestinal epithelial cells (IECs) disrupts mucosal regeneration and delays repair after injury. Here, we defined the role of HuR in regulating subcellular distribution of small guanosine triphosphatase Rac1 and investigated the implication of nucleophosmin (NPM) as a molecular chaperone in this process.

**Methods:**

Studies were conducted in intestinal epithelial tissue-specific HuR knockout (IE-HuR^-/-^) mice and cultured IEC-6 cells, derived from rat small intestinal crypts. Functions of HuR and NPM in vitro were investigated via their gene silencing and overexpression.

**Results:**

The abundance of cytoplasmic Rac1 in the small intestinal mucosa increased significantly in IE-HuR^-/-^ mice, although HuR deletion did not alter total Rac1 levels. HuR silencing in cultured IECs also increased the cytoplasmic Rac1 levels, without an effect on whole-cell Rac1 content. In addition, HuR deficiency in the intestinal epithelium decreased the levels of NPM in IE-HuR^-/-^ mice and cultured IECs. NPM physically interacted with Rac1 and formed the NPM/Rac1 complex. NPM silencing decreased the NPM/Rac1 association and inhibited nuclear accumulation of Rac1, along with an increase in cytoplasmic abundances of Rac1. In contrast, ectopically expressed NPM enhanced Rac1 nuclear translocation and restored Rac1 subcellular localization to near normal in HuR-deficient cells.

**Conclusions:**

These results indicate that HuR regulates Rac1 nucleocytoplasmic shuttling in the intestinal epithelium by altering NPM expression.

## Graphical abstract

**Figure:**
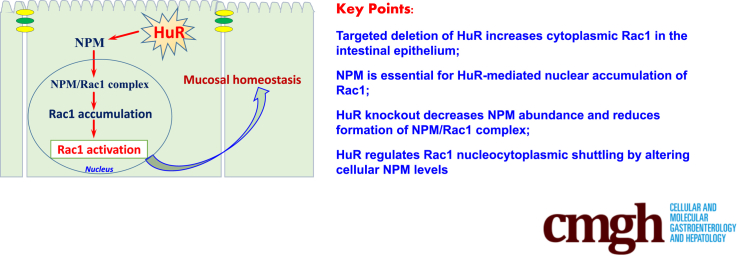

**Summary**

Rac1 plays an important role in the intestinal epithelium homeostasis. This study shows that RNA-binding protein HuR regulates nucleocytoplasmic trafficking of Rac1 by altering nucleophosmin. Targeted deletion of HuR decreases nucleophosmin, thus inhibiting the Rac1 nuclear accumulation.

The mammalian intestinal epithelium self-renews rapidly throughout the life of the organism,^1^, ^2^ and its homeostasis and integrity are tightly regulated by numerous factors including the small guanosine triphosphate (GTP)-binding protein Rac1. As a member of the Rho family of small GTPases, Rac1 and its homolog Rac2 bind to GTP and cycle between an inactive guanosine diphosphate–bound form and an active GTP-bound form through the activities of several guanine nucleotide-exchange factors and guanine nucleotide-activating proteins in physiological conditions.^3^ Rac1 plays fundamental roles in a wide variety of biological processes best known for its functions in cytoskeletal remodeling and control of cell migration.^3^, ^4^, ^5^ In addition to the cytosol and plasma membrane, Rac1 also modulates many nuclear functions, including transcriptional activation, cell-cycle progression, G2/M checkpoint activation, and response to DNA damage.^6^, ^7^, ^8^ The polybasic sequence of the Rac1 hypervariable region has been shown as a nuclear localization sequence that interacts with the nuclear import receptor karyopherin α2.^9^ The nuclear accumulation of Rac1 is tightly regulated and depends primarily on the cell cycle and different stimuli,^10^, ^11^ but the exact mechanisms underlying nucleocytoplasmic shuttling of Rac1 and its specifics inside the nucleus in the intestinal epithelium remain largely unknown.

The RNA-binding protein (RBP) HuR (encoded by the *Elavl1* gene) is highly expressed in the intestinal mucosa and acts as a key regulator of the epithelial homeostasis by regulating the stability and translation of target messenger RNAs (mRNAs).^12^, ^13^ HuR is among the most prominent translation and turnover regulatory RBP, and it interacts directly with Adenine Uracil-/Uracil-rich elements located in the 3’-untranslated regions (3’-UTRs) of labile mRNAs.^14^, ^15^ Upon binding to a target transcript, HuR stabilizes it, alters its translation, or performs both functions.^12^ For example, HuR associates with and stabilizes the mRNAs encoding p53, nucleophosmin (NPM), proto-oncogene, activating transcription factor-2 (ATF2), mitogen-activated protein kinase kinase-1, and Wingless-related integration site-co-receptor LDL receptor related protein 6 in intestinal epithelial cells (IECs),^12^, ^13^, ^16^, ^17^ and it also enhances translation of mRNAs encoding occludin, Cell division control protein 42, and cellular myelocytomatosis.^15^, ^18^, ^19^ Although constitutive HuR inactivation in vivo is lethal to embryos,^20^ intestinal epithelial tissue–specific deletion of HuR in mice inhibits regeneration of the intestinal mucosa, reduces tumor development, and delays repair of damaged mucosa induced by mesenteric ischemia/reperfusion in the small intestine and by dextran sulfate sodium in the colon.^13^, ^18^, ^21^ HuR also affects gene regulatory programs governing the gut epithelial functions by interplaying with different long noncoding RNAs (lncRNAs).^22^ HuR associates with lncRNA *SPRY4-IT1* to synergistically increase the stability and translation of mRNAs encoding tight junction proteins,^23^ and it binds to lncRNA *H19* to prevent the maturation of microRNA-675 from *H19*.^24^ More recently, HuR was found to regulate Paneth cell function by altering membrane localization of Toll-like receptor 2 via post-transcriptional control of Canopy FGF Signaling Regulator 3.^25^

NPM (also known as B23, encoded by the *NPM1* gene) is a phosphorylated and multifunctional protein that is implicated in several RNA regulatory mechanisms including transcription, ribosome assembly and biogenesis, mRNA stability and translation, and microRNA processing.^26^ NPM is localized predominantly in the nucleolus, but a proportion of the protein continuously shuttles between the nucleus and the cytoplasm.^27^, ^28^ NPM can bind to proteins containing nuclear localization signals for their import and functions as a molecular chaperone. Recently, NPM also was identified as a nuclear Rac1-interacting protein.^29^ Given the importance of HuR in gut epithelium homeostasis and its involvement in post-transcriptional control of NPM expression,^13^, ^18^, ^30^, ^31^ we sought to investigate the exact role of HuR in regulating subcellular distribution of Rac1 in the intestinal epithelium and further define the involvement of NPM in this process. The data presented herein show that HuR regulates nucleocytoplasmic trafficking of Rac1 through NPM, whereas targeted deletion of HuR in IECs enhances the cytoplasmic levels of Rac1 by decreasing NPM abundance. Because all HuR, NPM, and Rac1 are crucial for intestinal epithelium homeostasis,^13^, ^30^, ^32^ these findings provide a strong rationale for developing new therapeutic strategies directed at this pathway to preserve the epithelium integrity in patients with critical disorders.

## Results

### Intestinal Epithelium-Specific HuR Deletion Increases the Cytoplasmic Levels of Rac1

**Figure 1:**
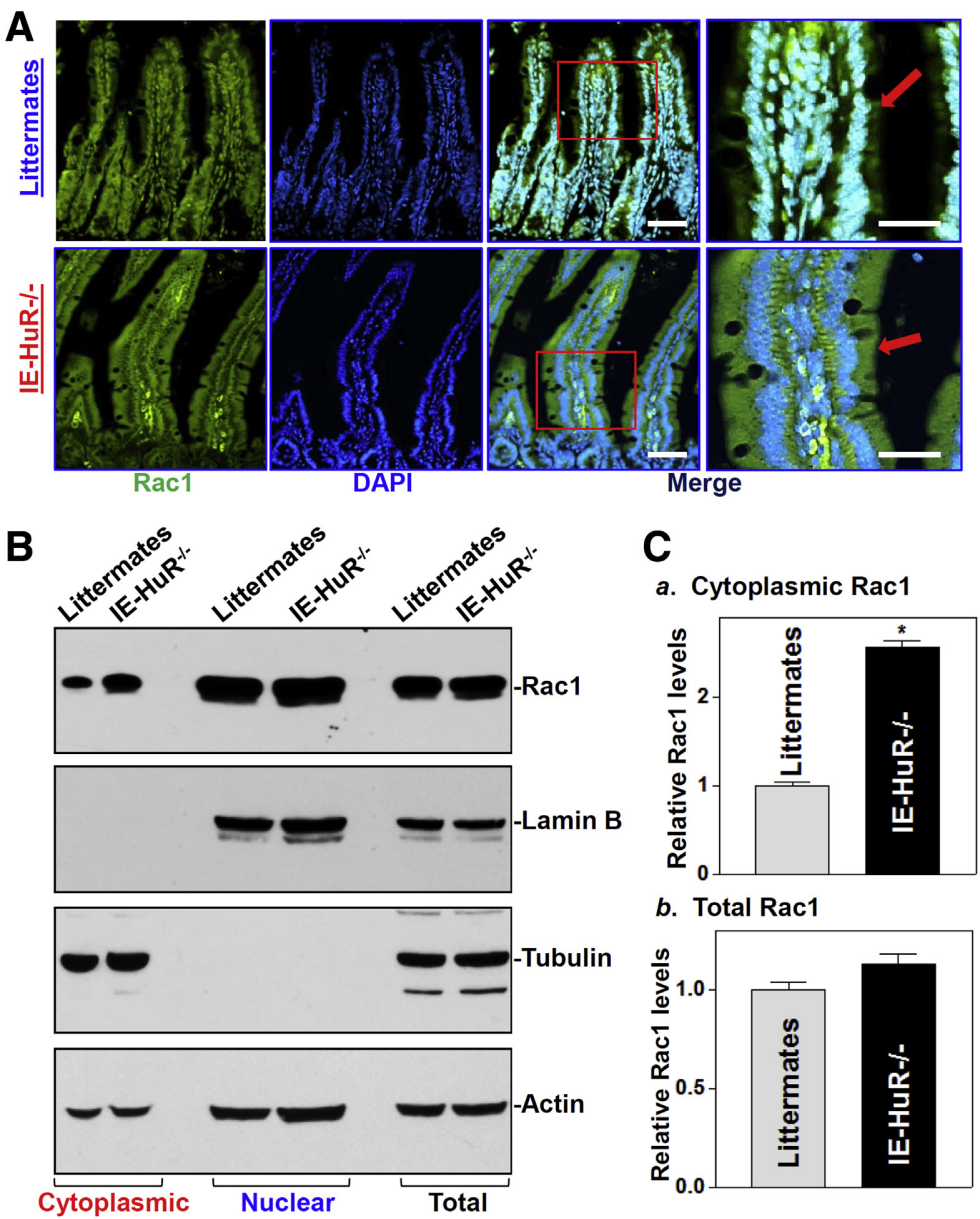
**Changes in subcellular distribution of Rac1 protein in the HuR-deficient intestinal epithelium in vivo.** (*A*) Immunohistochemical staining of Rac1 in small intestinal mucosa in control littermate and IE-HuR^-/-^ mice. Green, signals of Rac1; blue, nuclei stained by 4′,6-diamidino-2-phenylindole (DAPI). *Red arrows* point to Rac1 localization; *red box* define the area to be amplified. *Scale bar*: 50 μm. (*B*) Representative immunoblots in cytoplasmic (*left*), nuclear (*middle*), and total (*right*) Rac1 protein in small intestinal mucosa. Cytoplasmic, nuclear, and total proteins were isolated from the small intestinal mucosa and prepared for Western blot analysis. Equal loading in total proteins was monitored by actin; the quality and loading of the cytoplasmic and nuclear fractions were monitored and examined by β-tubulin and lamin B, respectively. Three experiments were performed that showed similar results. (*C*) Quantitative analysis derived from densitometric scans of immunoblots of cytoplasmic (*a*) and total Rac1 (*b*) as described in panel *B*. Values are the means ± SEM of 3 separate experiments. *P < .05 compared with littermates.

To begin to determine the involvement of HuR in the regulation of subcellular trafficking of Rac1 in the intestinal epithelium, we used intestinal epithelial tissue-specific HuR knockout (IE-HuR^-/-^) mice that were generated recently.^13^ As described in our previous studies,^13^, ^18^ HuR levels in the intestinal mucosa were undetectable in IE-HuR^-/-^ mice, but were at wild-type levels in other tissues and organs such as gastric mucosa, lung, liver, and pancreas (data not shown). HuR deletion inhibited the nuclear accumulation of Rac1 in the intestinal mucosa, as indicated by an increase in cytoplasmic Rac1 in IE-HuR^-/-^ mice relative to those observed in control littermates. Immunohistochemical staining assays showed that Rac1 immunoreactive signals in the intestinal mucosa were located mainly in the nucleus in control littermates, but these signals were localized predominantly within the cytoplasm of the intestinal epithelium in IE-HuR^-/-^ mice (Figure 1*A*). The changes in Rac1 subcellular distribution in the intestinal mucosa also were examined by Western immunoblot analysis and showed that the levels of cytoplasmic Rac1 in the HuR-deficient epithelium increased by approximately 2.5-fold (Figure 1*B* and *C*) compared with those in control littermates. Consistent with findings reported in our previous study,^18^ HuR deletion did not alter the total levels of Rac1 in the intestinal mucosa. Increased levels of cytoplasmic Rac1 abundance in the HuR-deficient intestinal epithelium was specific, because HuR deletion in IECs inhibited expression of Cdc42 and RhoB, increased RhoC, and did not affect subcellular distribution of these small GTP-binding proteins as shown previously.^18^ To monitor the quality and abundance of the cytoplasmic and nuclear fractions used in these studies, the levels of β-tubulin (a cytoplasmic protein) and lamin B (a nuclear protein) were examined and served as loading controls. Measurement of these markers showed that there was no contamination between cytoplasmic and nuclear fractions isolated from the intestinal mucosal tissues.

**Figure 2:**
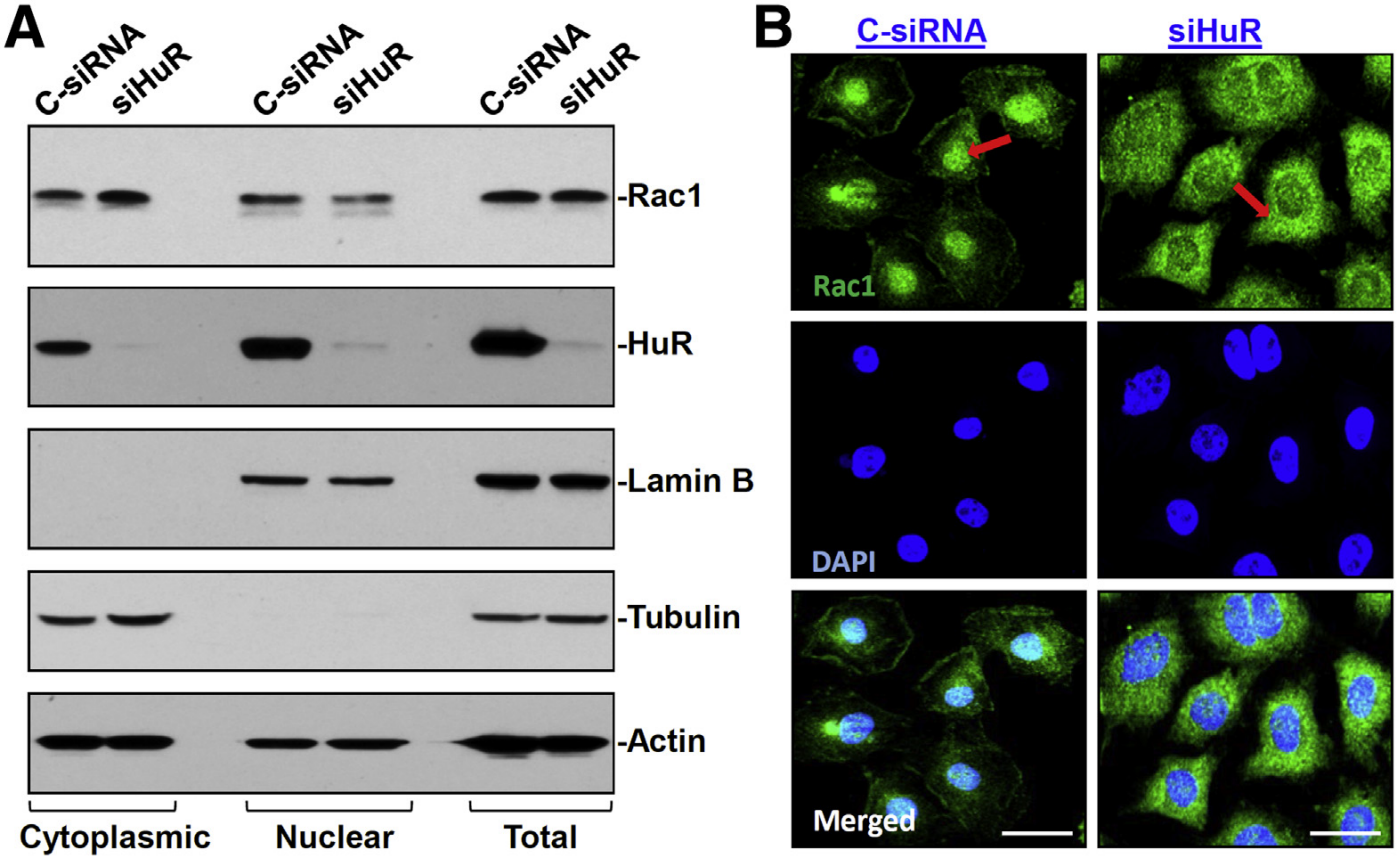
**HuR silencing increases cytoplasmic Rac1 abundance in cultured IEC-6 cells.** (*A*) Representative Western blots in cytoplasmic (*left*), nuclear (*middle*), and total (*right*) Rac1 protein in IEC-6 cells. After cells were transfected with siHuR or C-siRNA, whole-cell lysates were harvested 48 hours thereafter. Equal loading in cytoplasmic, nuclear, and total proteins were monitored by β-tubulin, lamin B, and actin, respectively. (*B*) Immunostaining of Rac1 in cells described in panel *A*. Cells were permeabilized and incubated with the anti-Rac1 antibody and then with anti-IgG antibody conjugated with Alexa Fluor. *Red arrows* point to Rac1 signals. Nuclei were stained with 4′,6-diamidino-2-phenylindole (DAPI). Green, signals of Rac1; blue, nuclear stained by DAPI. *Scale bar*: 5 μm. Three experiments were performed that showed similar results.

**Figure 3:**
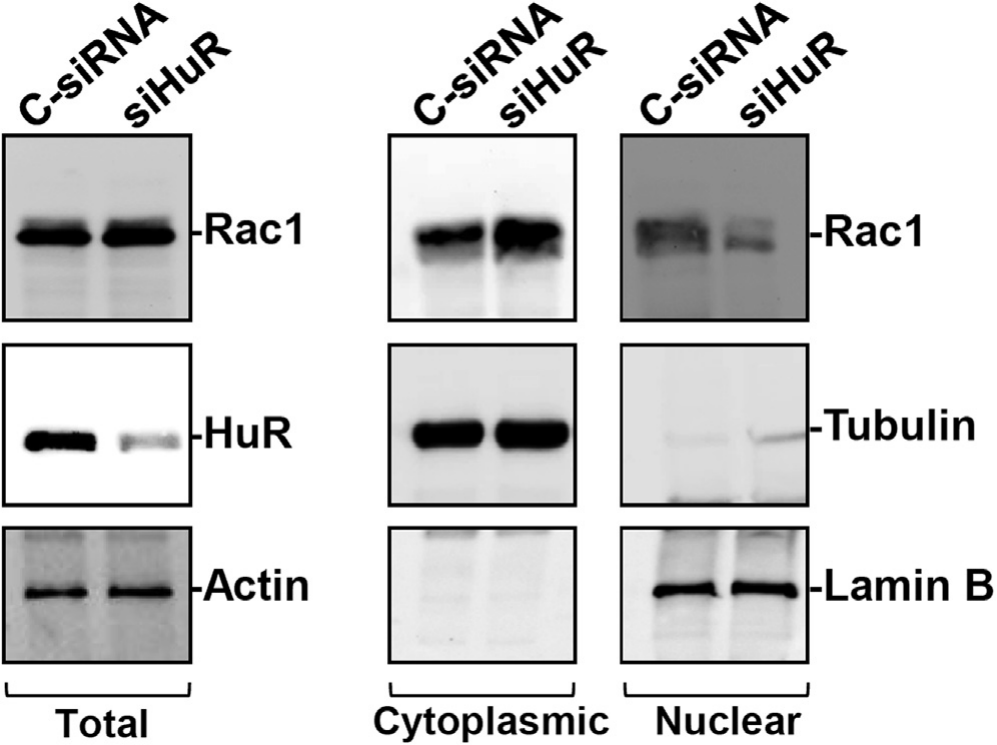
**HuR silencing increases cytoplasmic Rac1 abundance in cultured Caco-2 cells.** After cells were transfected with siHuR or C-siRNA, whole-cell lysates were harvested 48 hours thereafter. Levels of total, cytoplasmic, and nuclear Rac1 were examined by Western blot analysis. Equal loading in cytoplasmic, nuclear, and total proteins was monitored by β-tubulin, lamin B, and actin, respectively. Three experiments were performed that showed similar results.

Regulation of Rac1 subcellular trafficking by HuR was examined further in an in vitro system. As shown in Figure 2*A*, decreased levels of HuR by transfection with small interfering RNA targeting HuR also increased the level of cytoplasmic Rac1, along with a decrease in nuclear Rac1 abundance. Similar to the findings observed in IE-HuR^-/-^ mice, decreasing the levels of cellular HuR by transfection with siHuR did not alter total Rac1 levels in cultured IEC-6 cells. In a population of cells in which HuR was silenced, the levels of cytoplasmic Rac1 were increased by more than 2-fold when compared with those in cells transfected with control small interfering RNA (C-siRNA). As a positive control, HuR silencing dramatically decreased Cdc42 levels in IEC-6 cells (data not shown), similar to the findings described in our previous study.^18^ The levels of β-tubulin and lamin B also were examined and showed no contaminations between cytoplasmic and nuclear fractions used in this study. Consistent with Western blot results, the levels of cytoplasmic Rac1 immunostaining signals also increased significantly in cells transfected with siHuR compared with those observed in cells transfected with C-siRNA (Figure 2*B*). We also examined the influence of HuR on Rac1 subcellular distribution in human intestinal Caco-2 cells and showed that HuR silencing by transfection with siHuR increased the cytoplasmic levels of Rac1 without an effect on its total cellular abundance (Figure 3). In addition, transfection with either siHuR or C-siRNA did not affect cell viability, as examined by Trypan blue staining (data not shown). These data indicate that decreasing the levels of cellular HuR increases the cytoplasmic levels of Rac1 in the intestinal epithelium in in vivo and in vitro systems.

### HuR Stimulate NPM Expression and Enhance Formation of the NPM/Rac1 Complex

**Figure 4:**
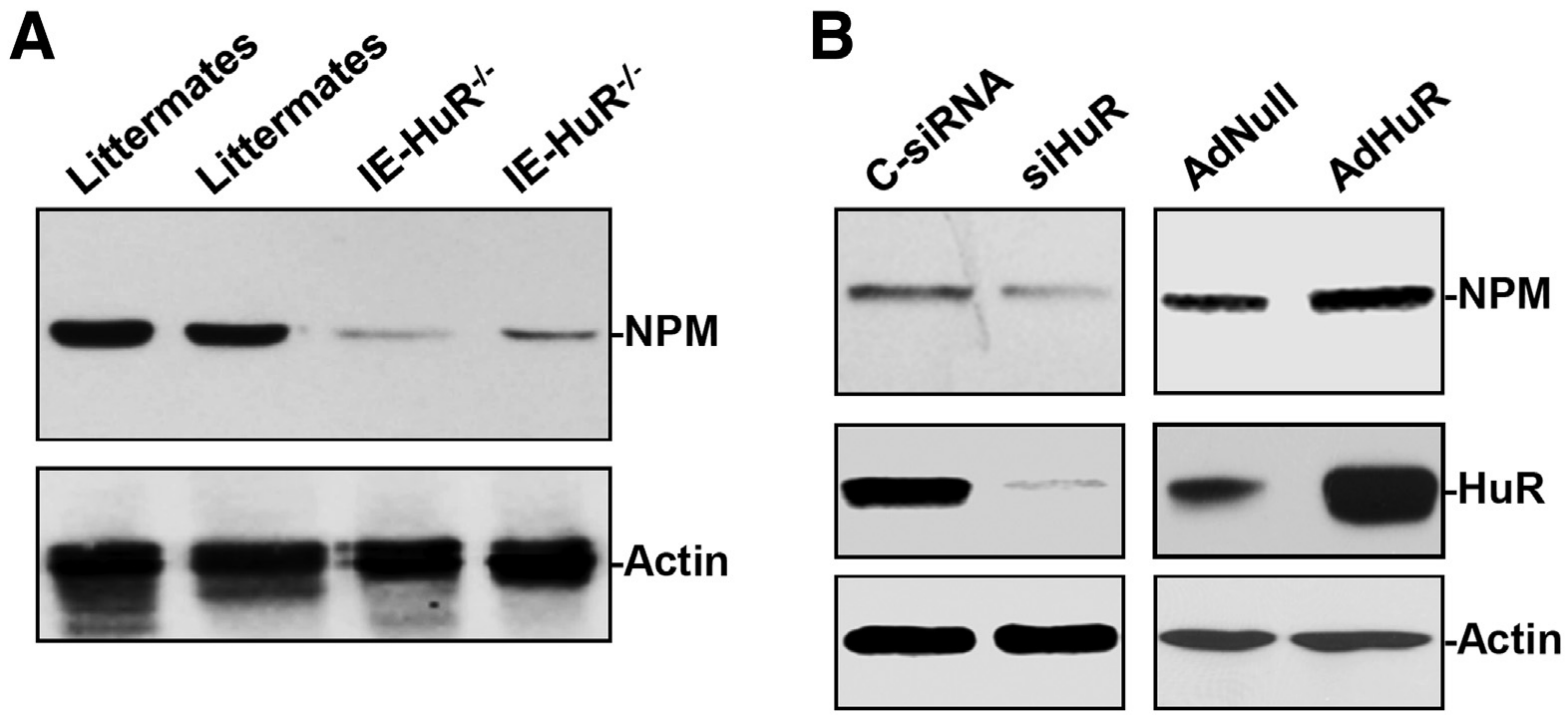
**HuR up-regulates NPM expression in the intestinal epithelium.** (*A*) Decreased levels of NPM protein in the HuR-deficient intestinal epithelium in mice. Total proteins were isolated from small intestinal mucosa, and the levels of NPM were measured by Western immunoblot analysis. Equal loading was monitored by actin. (*B*) Representative blots of NPM and HuR in IEC-6 cells transfected with siHuR or infected with the recombinant adenoviral vector encoding HuR cDNA (AdHuR) or adenoviral vector lacking HuR cDNA (Adnull) and analyzed 48 later. Three experiments were performed that showed similar results.

**Figure 5:**
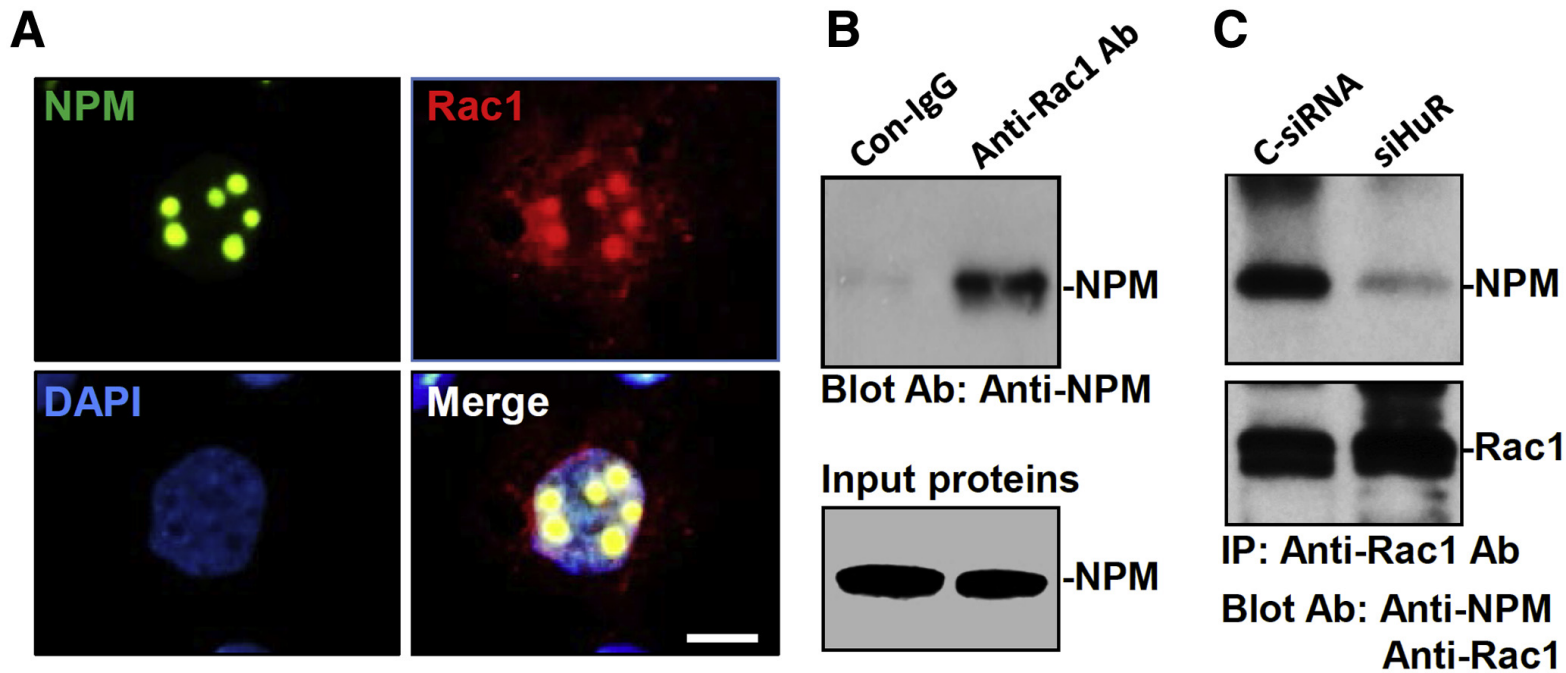
**NPM directly interacts with Rac1 in IEC-6 cells.** (*A*) Immunostaining of NPM and Rac1. Cells were permeabilized and incubated with either anti-NPM or anti-Rac1 antibody and then with anti-IgG antibody conjugated with Alexa Fluor. Nuclei were stained with 4′,6-diamidino-2-phenylindole (DAPI) (blue). Green, signals of NPM; red, signals of Rac1. *Scale bar*: 5 μm. (*B*) NPM/Rac1 association as measured immunoprecipitation (IP) assay using anti-Rac1 antibody (Ab). (*C*) HuR silencing decreases the levels of NPM/Rac1 complex without an effect on total Rac1 abundance. Cells were transfected with siHuR or C-siRNA, and total cell lysates were harvested 48 hours after the transfection. Three experiments were performed that showed similar results. Con, control.

To test the possibility that HuR regulates subcellular trafficking of Rac1 through NPM, we examined changes in the levels of NPM protein in the small intestinal mucosa after HuR deletion. In mice with ablated HuR, the intestinal mucosa showed a repression of NPM expression as indicated by decreased abundance of NPM protein (Figure 4*A*). The levels of NPM in the HuR-deficient epithelium decreased by approximately 85% (*n* = 3; *P* < .05) from those observed in control littermate mice. In cultured IEC-6 cells, HuR silencing by transfection with siHuR also decreased cellular NPM levels, whereas ectopically expressed HuR by infection with the recombinant adenoviral plasmids containing human HuR complementary DNA (cDNA) (AdHuR) increased the levels of NPM (Figure 4*B*). The levels of NPM in HuR-silenced cells decreased by approximately 70%, but its content in cells overexpressing HuR was more than 2-fold of that observed in cells infected with Adnull. Consistent with our previous findings,^31^ HuR was found to bind directly to the 3’-UTR of the *Npm* mRNA, and HuR silencing enhanced the degradation of *Npm* mRNA (data not shown). To define the role of NPM in the redistribution of Rac1 after HuR silencing, we examined the co-localization of NPM and Rac1 in IECs. As shown in Figure 5*A*, immunoreactive signals of NPM were localized only in the nucleolus in cultured IEC-6 cells. On the other hand, Rac1 was co-localized predominantly with NPM in the nucleolus, but some Rac1 immunostaining signals also were detectable in other areas of the nucleus and cytoplasm. We further examined the association of NPM with Rac1 by using immunoprecipitation assays. Whole-cell lysates were incubated with an antibody that recognized Rac1; after immunoprecipitation, the levels of NPM in the immunoprecipitation materials were examined by Western blot. As shown in Figure 5*B*, NPM physically interacted with Rac1 and formed the NPM/Rac1 complex in cultured IEC-6 cells. Moreover, decreasing the levels of cellular HuR by transfection with siHuR reduced the levels of NPM/Rac1 complex, although it did not alter the total Rac1 level (Figure 5*C*). These results indicate that HuR deletion inhibits NPM expression and decreases NPM/Rac1 interactions in IECs.

### NPM Is Essential for HuR-Mediated Nuclear Accumulation of Rac1

**Figure 6:**
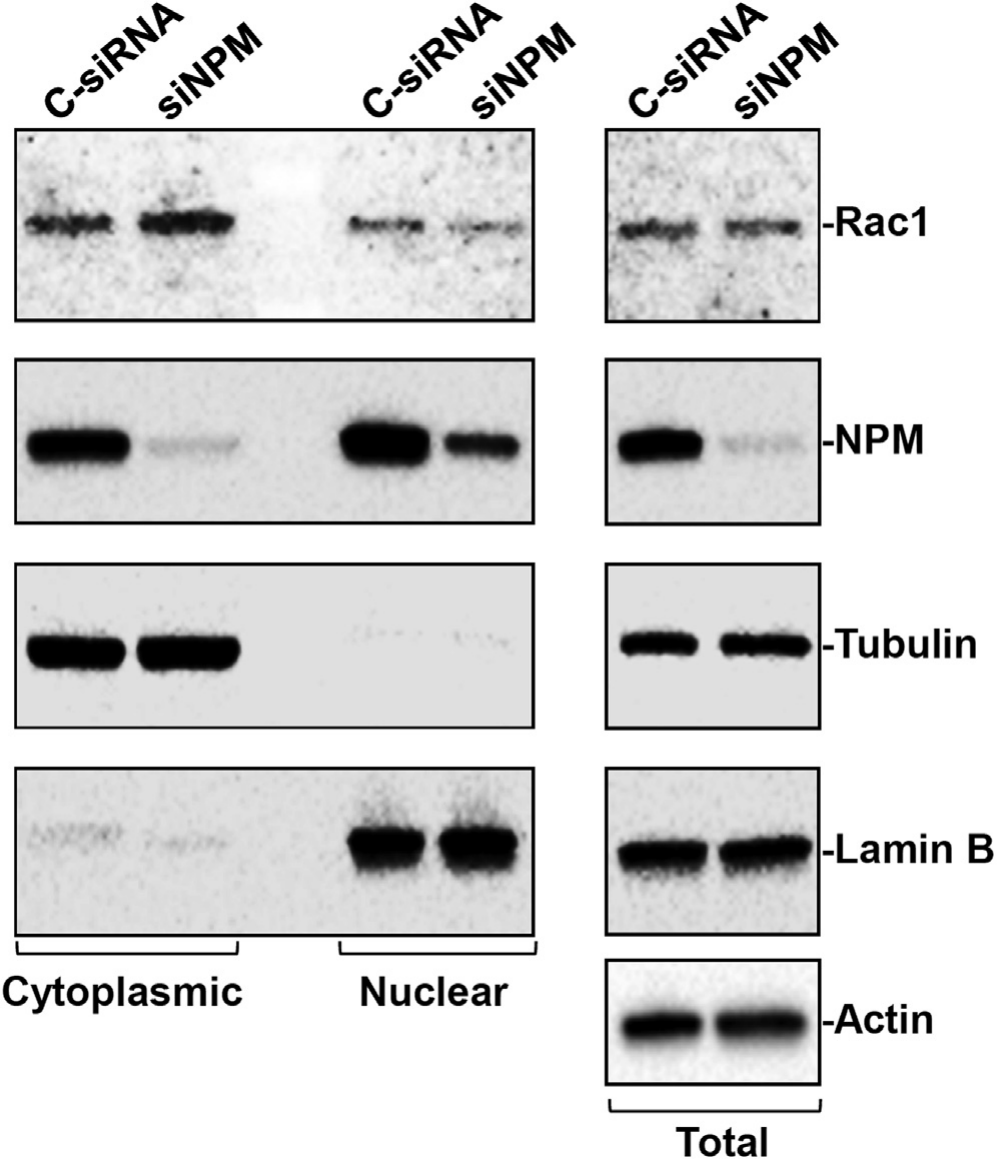
**NPM silencing increases cytoplasmic Rac1 levels in cultured IEC-6 cells.** After cells were transfected with siNPM or C-siRNA, whole-cell lysates were harvested 48 hours thereafter. Cytoplasmic (*left*), nuclear (*middle*), and total (*right*) proteins were prepared, and levels of Rac1 and NPM proteins were examined by Western blot analysis. Equal loading in cytoplasmic, nuclear, and total proteins was monitored by β-tubulin, lamin B, and actin, respectively. Three experiments were performed that showed similar results.

To investigate the function of NPM/Rac1 interaction in HuR-regulated Rac1 nuclear accumulation, we determined if manipulating the cellular NPM content, either decreased or increased, altered cytoplasmic Rac1 levels in control and HuR-deficient IECs. Forty-eight hours after transfection, the levels of total, cytoplasmic, and nuclear NPM decreased dramatically in cells transfected with small interfering NPM compared with cells transfected with C-siRNA (Figure 6). In fact, cytoplasmic NPM levels in siNPM-transfected cells were almost undetectable, and nuclear NPM decreased by more than approximately 70%. Importantly, decreasing the levels of cellular NPM by transfection with siNPM inhibited the nuclear accumulation of Rac1, as shown by an increase in cytoplasmic Rac1 abundance in NPM-silenced populations of IEC-6 cells. The levels of cytoplasmic Rac1 in siNPM-transfected cells were increased by approximately 2-fold compared with those in cells transfected with C-siRNA. β-tubulin and lamin B were examined to monitor the quality of cytoplasmic and nuclear fractions and between these subcellular fractions used in this study.

**Figure 7:**
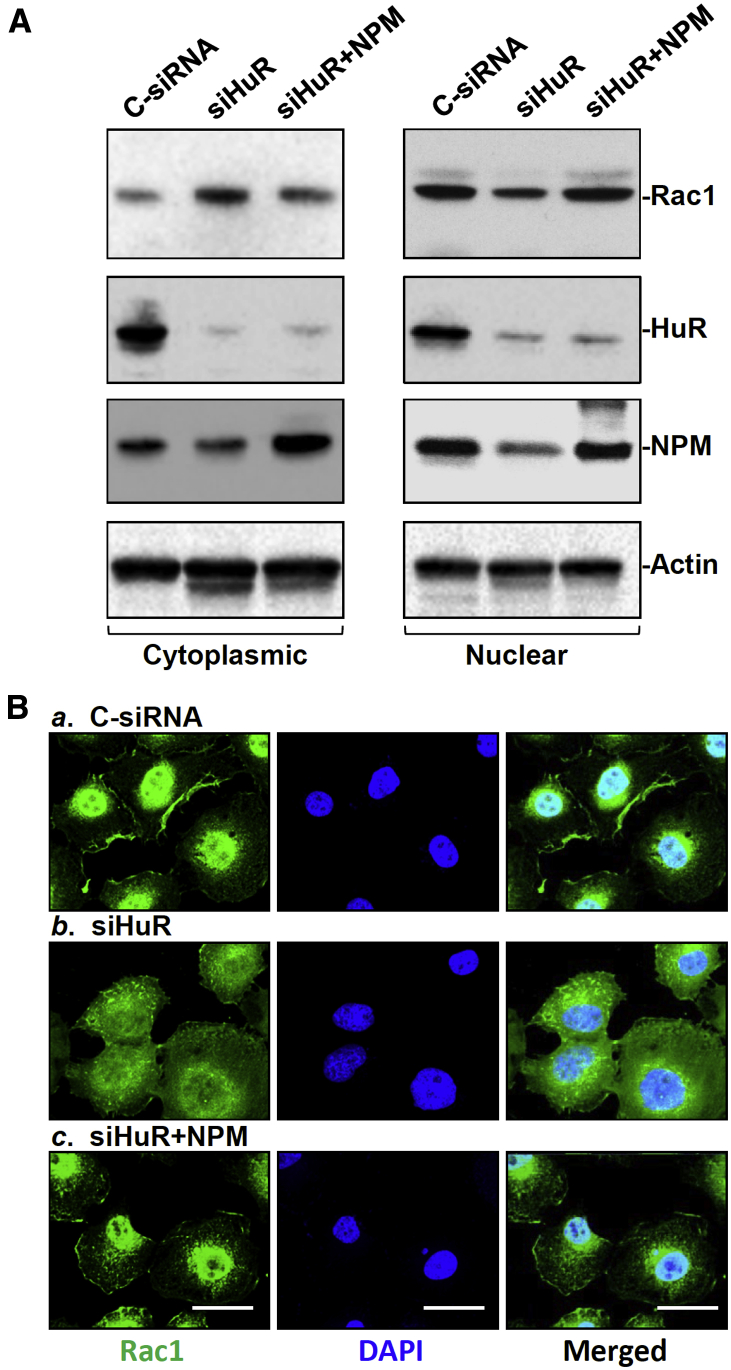
**Ectopically expressed NPM restores nuclear accumulation of Rac1 in the HuR-silenced cells.** (*A*) Representative blots of cytoplasmic Rac1, HuR, and NPM in cells transfected with siHuR alone or co-transfected with siHuR and NPM expression vectors. Cytoplasmic proteins were isolated and analyzed by Western immunoblot analysis. Equal loading was monitored by β-tubulin. (*B*) Immunostaining of Rac1 in cells described in panel *A*. (*a*.) Cells transfected with Control siRNA; (*b*.) cells transfected with siHuR; (*c*.) cells transfected with siHuR and NPM vector. Cells were incubated with the anti-Rac1 antibody and then with anti-IgG antibody conjugated with Alexa Fluor, whereas nuclei were stained with 4′,6-diamidino-2-phenylindole (DAPI). *Scale bar*: 5 μm. Green, signals of Rac1; blue, nuclear staining. Three experiments were performed that showed similar results.

Moreover, ectopically overexpressed NPM enhanced nuclear Rac1 translocation and restored Rac1 subcellular localization to near normal in HuR-deficient cells. As shown in Figure 7*A*, the levels of cytoplasmic Rac1 increased after HuR silencing, but this induction was prevented by ectopic overexpression of NPM. Although the levels of cytoplasmic Rac1 after the co-transfection with siHuR and NPM expression were still slightly higher than those observed in cells transfected with C-siRNA, it decreased by approximately 50% (*n* = 3; *P* < .05) from that observed in cells transfected with siHuR alone. Consistently, the nuclear levels of Rac1 in cells co-transfected with siHuR and NPM expression vector were returned to almost near-normal levels. Immunofluorescence staining further showed that HuR silencing by transfection with siHuR caused a dramatic increase in Rac1 immunostaining signals in the cytoplasm, which was significantly prevented by ectopic overexpression of NPM (Figure 7*B*). These results indicate that HuR regulates nucleocytoplasmic Rac1 trafficking via NPM in IEC-6 cells and that formation of the NPM/Rac1 complex is essential for Rac1 nuclear accumulation.

## Discussion

Rac1 is a multifunctional small GTPase and its subcellular distribution plays an important role in regulating its signaling output.^33^^,^^34^ Rac1 achieves differential regulation of signaling in different cellular contexts,^10^, ^29^ and nuclear translocation of Rac1 could be mediated by interacting with a Rac1-binding protein or by nuclear transport signals upstream of the Rac1 C-terminal domain.^10^, ^35^ Although the sequences responsible for Rac1 nuclear localization are well characterized,^36^ the exact mechanisms that control nucleocytoplasmic Rac1 shuttling is poorly understood. In this study, we show that the RBP HuR enhances nuclear accumulation of Rac1 in the intestinal epithelium by increasing cellular abundance of NPM, which acts as a Rac1 nuclear interactor. In contrast, conditional HuR deletion in IECs reduces NPM levels and NPM/Rac1 interactions, thus inhibiting nuclear translocation of Rac1. The present studies implicate HuR in the control of subcellular trafficking of Rac1 in the intestinal epithelium through modulation of NPM expression, thereby advancing our understanding of HuR biological functions and the regulation of Rac1 subcellular distribution.

Several recent studies using mice with HuR transgene or ablated HuR in diverse tissue/organ cell types have improved our understanding of physiological roles of HuR in mammals.^12^ For examples, overexpression of HuR in mouse macrophages alters the translation of selective inflammatory mRNAs,^37^ and fertility is compromised in mice overexpressing HuR in testis.^38^ On the other hand, myeloid deletion of HuR exacerbates the production of proinflammatory cytokines and increases the sensitivity to acute inflammatory reactions such as endotoxemia,^39^ and HuR deletion in germ cells leads to male sterility, but not female sterility.^40^ HuR also is essential for maintaining hematopoietic stem cells, the selection and chemotaxis of T cells, and B-cell development and activation/differentiation after antigen encounter.^41^, ^42^, ^43^ Results from our in vivo and in vitro studies further show that HuR deletion in IECs enhances the cytoplasmic Rac1 abundance, although this intervention does not alter whole-cell Rac1 levels. We show the importance of HuR in regulating Rac1 subcellular localization in the intestinal epithelium. Because Rac1 is crucial for nuclear cytoskeletal polymerization and nuclear structure organization,^29^ our findings suggest that the stimulation of nuclear Rac1 accumulation by HuR plays a critical role in different intestinal epithelial functions.

Our results also indicate that HuR-regulated nucleocytoplasmic Rac1 shuttling is mediated by NPM protein in the intestinal epithelium. Targeted HuR deletion in IECs decreased the NPM levels in the small intestinal mucosa, which was associated with an increase in the cytoplasmic Rac1. In studies in vitro, HuR silencing reduced the levels of cellular NPM/Rac1 complex and thus increased cytoplasmic Rac1 abundance by reducing its nuclear transport, whereas ectopically expressed NPM restored Rac1 subcellular organization to near normal in HuR-silenced cells. NPM initially was identified as a nucleolar chaperone protein that constantly shuttles between the nucleolus and cytoplasm, which is involved in diverse biological functions, including genomic stability and tumorigenesis, ribosome biogenesis, and centrosome duplication.^44^, ^45^, ^46^, ^47^ In this regard, NPM interacts with ATF5 via its C-terminal region where nucleolar localization signal of NPM resides, and this interaction increases ATF5 nucleolar accumulation.^48^ Recent studies further showed that NPM also continuously shuttles between the nucleus and cytoplasm, through which it is implicated in different nuclear processes by interacting with several binding proteins.^3^, ^6^ NPM was found to act as a Rac1 chaperone in the nucleus and regulate Rac1 subcellular localization.^29^ Control of Rac1 nuclear accumulation by NPM leads to 2 functional consequences, altering the nuclear membrane organization and regulating the cytoplasmic ratio of Rac1 and Rho, thus modulating cell migration and tumor invasion. In cultured Fibroblast like cell line COS7 (COS7) and Human cervical cancer cell line (HeLa) cells, however, NPM favors Rac1 nuclear export because NPM silencing increases the levels of nuclear Rac1.^29^ The exact reasons underlying the discrepancy of our findings in the intestinal epithelium with the observations in cultured COS7 and HeLa cells remain unknown, but these results suggest that the function of NPM in the regulation of Rac1 subcellular distribution is cell-type–dependent and can be regulated by many other intracellular and extracellular factors. As shown in Figure 5*A*, NPM is only localized in the nucleus in IEC-6 cells. Consistent with our current findings, NPM also physically interacts with Rac1 in COS7 and HeLa cells. It is unclear how NPM silencing modulates nuclear Rac1 levels differentially in different types of cells. Clearly, more studies, particularly in vivo experiments using genetically modified animal models, are needed to fully investigate the exact role of NPM in the control of nucleocytoplasmic Rac1 shuttling and its mechanism.

Our previous studies have shown that HuR in cultured IECs regulates expression of several proliferation/apoptosis-associated genes including NPM.^13^, ^18^, ^21^, ^30^ HuR directly interacts with and stabilizes the *Npm* mRNA through its 3’-UTR rather than its 5’-UTR and coding region, whereas decreasing the levels of cellular HuR by transfection with siHuR enhances degradation of the *Npm* mRNA in cultured IEC-6 cells.^31^ HuR is localized predominantly in the nucleus in unstimulated cells, but it translocates to the cytoplasm rapidly in response to various stimuli such as polyamine depletion.^31^, ^49^ HuR binding to the mRNAs encoding occludin and c-Myc requires HuR phosphorylation induced by Checkpoint kinase 2 (Chk2) kinase,^19^, ^50^ but HuR association with the *Npm* mRNA is dependent primarily on the presence of cytoplasmic HuR regardless of its phosphorylation.^31^ Depletion of cellular polyamine increases cytoplasmic HuR levels and enhances HuR/*Npm* mRNA association, although it inhibits Chk2-mediated HuR phosphorylation. In contrast, HuR binding to the *occludin* and *c-myc* mRNAs decreases dramatically in polyamine-deficient IECs because of inhibition of Chk2 kinase and the subsequent decrease in HuR phosphorylation.^50^, ^51^ The mechanism by which HuR regulates mRNA stability and translation remains largely unknown, but several studies have shown that HuR blocks translocation of its target mRNAs to processing body and stress granules, where mRNAs are sorted for degradation and translation repression.^52^, ^53^

Although the exact biological function of nuclear Rac1 in the intestinal epithelium remains to be fully investigated, our results provide novel evidence showing that HuR is necessary for nuclear accumulation of Rac1 through up-regulation of NPM. Several studies have shown that active Rac1 in the nucleus activates extracellular-signal-regulated kinase 1/2 signaling and the G2/M checkpoint,^7^, ^8^ promotes cell division,^10^ and is required for mammary epithelial stem cell self-renewal.^11^ We have shown that HuR deletion in IECs leads to the intestinal mucosal atrophy and also delays mucosal healing after acute injury.^13^, ^18^, ^21^ The current findings suggest that reduction in nuclear Rac1 accumulation in HuR-deficient intestinal epithelium also may contribute to the process by which HuR deletion inhibits intestinal epithelial regeneration. In sum, our results indicate that HuR is a novel regulator of Rac1 nucleocytoplasmic shuttling and that HuR is essential for the maintenance of intestinal epithelium hemostasis and integrity, at least partially by altering the subcellular distribution of Rac1 via post-transcriptional control of NPM expression.

## Materials and Methods

### Chemicals and Cell Cultures

The culture medium and fetal bovine serum were purchased from Invitrogen (Carlsbad, CA) and biochemicals were from Sigma (St. Louis, MO). The antibodies recognizing NPM (cat. 3542; Cell Signaling Technologies, CST, Danvers, MA), HuR (cat. SC-5261; Santa Cruz Biotechnology, Santa Cruz, CA), Rac1 (cat. 33186; Abcam, Cambridge, UK), lamin B (cat. SC-6216; Santa Cruz Biotechnology), β-tubulin (cat. 3873; Cell Signaling Technologies), and actin were obtained from Santa Cruz Biotechnology (cat. SC-58673; Santa Cruz Biotechnology), and the secondary antibody conjugated to horseradish peroxidase was from Sigma. All antibodies used in this study were thoroughly validated for species specificity. Antibody dilutions used for Western blots of HuR, NPM, Rac1, lamin B, β-tubulin, and actin were 1:1000 (first antibody) and 1:2000 (second antibody), respectively, whereas antibody dilutions for immunostaining were 1:200 (first) and 1:2000 (second). Relative protein levels were analyzed using the Bio-Rad (Hercules, CA) Chemidoc and XRS system equipped with Image lab software (version 4.1). We also used the Quantity Tool to determine the band intensity volume; the values were normalized with internal loading control actin. The IEC-6 cells, derived from normal rat intestinal crypt cells, were purchased from American Type Culture Collection (Manassas, VA) at passage 13 and maintained under standard culture conditions.^54^ Passages 15–20 were used in experiments, and there were no significant changes of biological function and characterization of IEC-6 cells at passages 15–20.^2^, ^52^

### Animal Studies

All animal experiments were performed in accordance with National Institutes of Health guidelines and were approved by the Institutional Animal Care and Use Committee of the University of Maryland School of Medicine and the Baltimore Veterans Affairs Hospital. The HuR^flox/flox^ (HuR^fl/fl^) and villin-Cre mice were purchased from the Jackson Laboratory (Bar Harbor, ME), and IE-HuR^-/-^ mice were generated by crossing the HuR^fl/fl^ mouse with the villin-Cre mouse as described in our previous studies.^13^, ^18^ HuR^fl/fl^-Cre^-^ mice served as littermate controls. Both IE-HuR^-/-^ mice and control littermates were housed and handled in a specific pathogen–free breeding barrier and cared for by trained technicians and veterinarians. Animals were deprived of food, but allowed free access to tap water for 24 hours before experiments. Two 4-cm small intestinal segments taken 0.5-cm distal to the ligament of Trietz were removed in each animal. One segment was for chemical analysis, and the other segment was for histologic examination. The intestinal mucosal tissues were scraped with a glass slide for protein extraction.^2^, ^55^

### Plasmid Construction

AdHuR were constructed using the Adeno-X Expression System (Clontech, Mountain View, CA) as described previously.^21^ Briefly, the full-length cDNA of human wild-type HuR was cloned into the empty backbone vector (pShuttle) by digesting the BamHI/HindIII and ligating the resultant fragments into the XbaI site of the pShuttle vector.^16^ adenoviral construction vector (pAdeno-HuR) was constructed by digesting the pShuttle construct with PI-SceI/I-CeuI and ligating the resultant fragment into the PI-SceI/I-CeuI sites of the pAdeno-X adenoviral vector. Recombinant adenoviral plasmids were packaged into infectious adenoviral particles by transfecting human embryonic kidney–293 cells using LipofectAMINE Plus reagent (Gibco-Bethesda Res Lab, Gaithersburg, MD). Titers of the adenoviral stock were determined by standard plaque-forming assay. Recombinant adenoviruses were screened for the expression of the introduced gene by Western blot analysis using an anti-HuR antibody. pAdeno-X, which was the recombinant replication-incompetent adenovirus carrying no HuR cDNA insert (Adnull), was grown and purified as described earlier and served as a control adenovirus. Cells were infected with AdHuR or Adnull, and expression of HuR was assayed at different times after the infection. The NPM expression vectors were purchased from (OriGene Technologies, Rockville, MD), and transient transfections were conducted using the LipofectAMINE reagent as recommended by the manufacturer.

### RNA Interference

HuR and NPM were silenced by transfection with specific siRNA as described.^53^ The siRNAs specifically targeting HuR mRNA (siHuR) or NPM mRNA (siNPM) and C-siRNA were purchased from Dharmacon (Lafayette, CO). For each 60-mm cell culture dish, 15 μL of the 20 μmol/L stock duplex siHuR, siNPM, or C-siRNA was used. Forty-eight hours after transfection using LipofectAMINE, cells were harvested for various analyses.

### Immunofluorescence Staining

The immunofluorescence staining procedure was performed according to the method described in our previous publications.^56^ For experiments using mucosal tissue samples from mice, dissected and opened intestines were mounted onto a solid surface and fixed in formalin and paraffin. Sections (5-μm thick) were prepared for immunofluorescence staining. For studies in cultured IEC-6 cells, the monolayers of control and cells transfected with various siRNAs or NPM expression vectors were fixed in 3.7% formaldehyde in phosphate-buffered saline and rehydrated. All slides were incubated with the primary antibody against Rac1 in the block buffer at a concentration of 1:300 dilution at 4ºC overnight and then incubated with a secondary antibody conjugated with Alexa Fluor–594 (Molecular Probes, Eugene, OR) for 2 hours at room temperature. After rinsing 3 times, the slides were incubated with 4′,6-diamidino-2-phenylindole (Molecular Probes) at a concentration of 1 μmol/L for 10 minutes to stain cell nuclei. Finally, the slides were washed, mounted, and viewed through a Zeiss (Oberkochen, Germany) confocal microscope (model LSM710). Images were processed using Photoshop software (Adobe, San Jose, CA).

### Preparation of Cytoplasmic and Nuclear Proteins, Immunoprecipitation, and Western Blot Analysis

Cytoplasmic and nuclear proteins were prepared using the Protein Extraction kit (Life Technologies Corporation, Grand Island, NY) as described previously,^31^ and the protein contents in different preparations were measured using the Bradford method. For studies of immunoprecipitation, equal amounts of proteins for each sample were incubated with the specific antibody against Rac1 at 4ºC for 3 hours, and protein A/G-PLUS-Agarose (Santa Cruz Biotechnologies, Santa Cruz, CA) was added and incubated overnight at 4ºC. The precipitates were washed fully, and the beads were resuspended in sodium dodecyl sulfate sample buffer. Samples were boiled for 5 minutes and then subjected to electrophoresis on 7.5% acrylamide gels. After the blots were incubated with primary and secondary antibodies, immunocomplexes were developed using chemiluminescence.

### Statistical Analysis

All values were expressed as the means ± SEM from 3 separate experiments (*n* = 3). Unpaired, 2-tailed Student *t* tests were used when indicated, with *P* < .05 considered statistically significant. When assessing multiple groups, 1-way analysis of variance was used with the Tukey post hoc test.^57^ The statistical software used was GraphPad Instat Prism 5 (San Diego, CA). For nonparametric analysis rank comparison, the Kruskal–Wallis test was conducted.
